# Counter Statement of Wm. R. Warner & Co.

**Published:** 1878-03-20

**Authors:** 


COUNTER STATEMENT *by William R. Warner & Co., in reply to an article from Bullock & Crenshaw in the January number of the Southern Medical Record.*

In an article, published in a previous number of the "Southern Medical Record," written in the interest of the firm of Bullock & Crenshaw, with other statements, claim was made to priority for them in the manufacture of sugar-coated pills. We presumed that these statements were furnished the writer, and have since been informed that such was the case; they said the article was unsolicited. To the claim of priority we made a reply in the November number of the same journal, denying its accuracy. In the January number a rejoinder appeared, in which it is acknowledged that "W. R. Warner supplied them with sugar-coated pills at a stipulated price;" but set up a justification of their claim by the fact that the pills were labelled and sold as their manufacture, and that he was not known in the matter. We have the original books of entry to show the debits made against this house for pills so furnished, which were manufactured at the store of W. R. Warner, in a remote part of the city; and visited but once by one member of that firm, about the close of the eighth year. How does this compare with their statement, that no other firm or individual was similarly engaged in the State of Pennsylvania? This is a question, however, of little importance contrasted with the endeavor on their part to lessen the merit of the regular Centennial Judges, by casting an insinuation on account of the appointment of the supplementary judges. Director General Goshorn says:

"Under the system of awards adopted by the Centennial Commission, the governments represented in the Exhibition designated judges selected for their *special knowledge of the subjects assigned them* to report upon, including some of the most eminent scientists in the world. The character of the judges, and *the careful attention which they gave to their examinations*, afford assurance that their reports generally will embody a record of the latest advances made in the industries submitted to their inspection.

The fling at Dr. J. H. Thompson, one of the judges, in which he is styled as being "so active," has no meaning, or else it is an insinuation with which he can deal in his own way. We delayed this answer, in the hope of hearing from Professor Thompson, who is now in Europe, and for the purpose of taking some evidence which we had intended using in this connection. Supplementary judges were not appointed for any fault or inexcusable omission on the part of the primary judges, but for the purpose of looking after exhibits that had been overlooked or not classified, and for the purpose of hearing appeals. This led to the very general distribution of awards. The primary judges had discriminated in favor of the most meritorious and had taken great pains in their examinations. In our case, the laboratory was visited, processes examined, samples of pills taken and submitted to chemical analysis, and their relative solubility tested. This group of judges comprised men, among the most eminent in the professions of medicine and chemistry. The language of their award reads as follows:

"The sugar coated pills of Wm. R. Warner & Co., are soluble, reliable and unsurpassed in the perfection of sugar-coating, thorough composition and accurate sub-division. The pills of phosphorus are worthy of special notice. The element is thoroughly diffused and sub-divided, yet perfectly protected from oxidation."

The supplementary group of judges consisted of three gentlemen, eminent in their respective professions and whom we highly honor and respect. One is a surgeon, one a naturalist and one a machinist. When asked to examine our exhibit they refused, saying, it had been examined; but, they made an award to Bullock and Crenshaw, the language of which will be seen in the official catalogue of awards, as follows: "Commended for superior workmanship, quality and fitness for purposes intended." They, B and C., publish that they "received a diploma and medal for superiority of quality and finish," and on the adjoining page, "superiority of finish and purity of ingredients."

On this comment is unnecessary. With the exception of a few special gold medals, for the Corliss Engine, etc., there was but one grade of award, consisting of a bronze medal and a diploma. The text of the award constitutes its value, and it is generally acknowledged that the awards made by the primary judges are of greater value and importance.

They also lay claim to language never used, in the following, taken from a calendar now before us:

"A paper, read at the annual meeting of the American Pharmaceutical Association, Boston, 1875, asserts that careful experiments made with fair samples of the best pills in the market, B. & C.'s sugar coated having been selected, that of the three forms of the ready-made pills of the day in general use, sugar coated pills are to be prepared in point of solubility."

Reference to the article does not show any superiority of their sugar coated pills over any others similarly coated; three makes having been used, and no names mentioned. The experiments were simply directed to the question as to the superior merits of gelatin or sugar, as a material for coating. We cite this as an additional illustration of their disingenuousness.

1t WM. R. WARNER & Co.

